# Introducing an Emergency Department Electronic Handbook to Junior Doctors New to Emergency Medicine

**DOI:** 10.7759/cureus.66313

**Published:** 2024-08-06

**Authors:** Udara Wickramanayake, Ahmed Arrayeh, Venoth Arulvasan, Bhaskar Sarvesh, Charles Nwankpa, Ravindu Hewagamage, Ayesha Malik, Angelo Giubileo

**Affiliations:** 1 Emergency Medicine, Queen Elizabeth Hospital King's Lynn NHS Foundation Trust, King's Lynn, GBR; 2 Medicine, University of East Anglia, King's Lynn, GBR; 3 Information Technology, Queen Elizabeth Hospital King's Lynn NHS Foundation Trust, King's Lynn, GBR

**Keywords:** confidence, junior doctor induction, induction satisfaction, induction handbook, ed e-handbook, king's lynn, queen elizabeth hospital, knowledge and confidence, ed induction, handbook

## Abstract

Background

The transition of junior doctors into working in the emergency department (ED) in the United Kingdom often poses challenges in adapting to new hospital systems and protocols. To address this issue at Queen Elizabeth Hospital, King’s Lynn (QEHKL), a quality improvement project (QIP) was undertaken to develop an electronic ED handbook with the primary aim of enhancing the confidence and knowledge of newly appointed doctors during their ED rotation. This electronic handbook serves as a comprehensive repository for vital medical protocols, guidelines, and trust referral pathways, offering an easily accessible resource for junior doctors.

Objectives

The primary objective of this study was to determine whether there was an improvement in the confidence and knowledge of ED junior doctors following the introduction of the Electronic ED Handbook. The secondary objectives were to determine whether introducing the ED Handbook increased the overall satisfaction rating of the content of the ED Junior Doctor Induction program and assess the level of recommendation for the ED Handbook among the doctors for inclusion in future ED inductions.

Method

The QIP was designed using the Model for Improvement framework, Plan, Do, Study, Act (PSDA). The aims were designed to be Specific, Measurable, Achievable, Relevant, and Time-bound (SMART). Pre- and post-intervention surveys were conducted for comparison before and after the ED Handbook was introduced.

Results

Regarding the confidence of junior doctors to proceed into their new roles, the responses of “quite confident” or “very confident” increased from 77.8% (before) to 100% (after the ED Handbook introduction). One hundred percent of the responders found the ED Handbook to be either “very useful” or “extremely useful” in increasing their confidence and knowledge in the first month of their ED rotation. The satisfaction rating of “excellent” for the content of the ED Junior Doctor Induction program increased from 55.5% to 66.7%. One hundred percent of the responders recommended the inclusion of the ED Handbook for future inductions.

Conclusion and recommendations

Comparing the results from the pre- and post-intervention surveys shows a significant improvement in the confidence and knowledge of ED junior doctors following the introduction of the Electronic ED Handbook. The handbook was formally endorsed by the ED clinical governance team as an integral component of the ED induction process, aiding junior doctors in making a seamless transition into their new roles in emergency medicine. This study emphasizes the importance of utilizing digital resources to improve the confidence and knowledge of junior doctors and recommends the continued use of the handbook in future induction programs.

## Introduction

Doctors new to a particular emergency department (ED) often struggle for months with navigating a new hospital system. To help mitigate this issue at Queen Elizabeth Hospital (King’s Lynn, UK), we have initiated a quality improvement project by creating the ED Handbook with the aim of increasing the confidence and knowledge of our new doctors in their first month of ED rotation. It is a compilation of the most important medical protocols, guidelines, and referral pathways - all in one electronic booklet, easily accessible on any smartphone.

This study will explore the need for this intervention by reviewing the literature on its positive effects observed in previous studies. Emergency department juniors may suffer a lack of confidence in making decisions about the care of patients in a high-pressure environment. This can potentially be worsened by the lack of senior supervision and adequate inductions [1]. In addition, junior doctor inductions can often be disorganized, inconsistent, and/or lacking in useful content [2]. They can also be too short and/or anxiety-inducing [3].

Current evidence supports that inductions are important for work productivity, confidence of trainees, and patient safety levels [4]. The delivery of inductions can vary from lectures and handouts to informal chats. This could result in differences in the quality of inductions across different trusts [4]. Kilminster et al. noted that they can be disorganized or inconsistent [5]. The General Medical Council (GMC) in the 2018 National Training Survey found that inadequate inductions could lead to challenges in the continuity of care of patients [6].

In 2018 the General Medical Council (GMC) Emergency Medicine (EM) training program had one of the highest percentages (33%) for ratings of “neither good/poor,” “poor,” or “very poor” from their trainees [6]. In the 2022 survey, EM trainees had the highest percentages of “very heavy” or “heavy” workload (77%) and risk of burnout (32%) compared to all the other specialties [7]. This highlights the importance of adequate inductions and sustained effective senior support.

Thomas et al. introduced an induction booklet and assessed the ability of junior doctors to understand work rotations, request investigations, and find important referral numbers [8]. Pre- and post-induction booklet surveys showed a positive impact of introducing the booklet on all mentioned tasks. However, the term junior doctor can refer to a Foundation Year 1 (FY1) doctor all the way up to a final year higher specialty trainee [9]. Therefore, there can be large variability in the level of experience from one junior doctor to the next. The paper does not specify or stratify the grades of the junior doctors. Dave et al. introduced a structured hepatopancreatic-biliary (HPB) surgery induction booklet for FY1 doctors [10]. They found significant improvements in both their knowledge and confidence in the management of acutely unwell HPB patients.

Davies et al. indicated that gaps in knowledge of incoming junior doctors to a department are partly a result of poor handover of information from the juniors who are rotating out [11]. They introduced a junior doctor handbook which was unique in having a “Ward Transition Form” that contained information about current patients and instructions for pending tasks. After the intervention, 70% responded that they found the handbook to be useful for their rotation. Forty-seven percent felt they were more efficient in their work and 67% found it helped them understand their role.

Clare et al. introduced an app called “Induction” (London, UK: Induction Healthcare) for referrals and bleep numbers [12]. Prior to introducing the app, 54% of juniors found challenges with requesting investigations and 100% found it challenging to find guidelines. Post-intervention surveys saw the figures reduce to 14.3% and 15.4%, respectively.

In NHS Tayside, a junior doctor's handbook for induction was introduced to tackle the problem of unreliable handovers between doctors rotating out and those rotating in. Seventy percent of the junior doctors indicated that the handbook improved their efficiency as new doctors at the FY1 level [13].

Similarly in Black Country Healthcare NHS Foundation Trust, a junior doctor psychiatric induction booklet was introduced [14], as a response to the decline in patient safety outcomes associated with the end-of-year changeover of doctors [15]. Ninety-five percent of respondents felt that the contents of the booklet were either satisfactory or very satisfactory. Ninety-five percent either agreed or strongly agreed that the booklet helped improve the delivery of safe patient care.

At NHS Greater Glasgow and Clyde, they used a combination of practical face-to-face demonstrations, clinical scenarios, and prescribing tasks, supplemented by written materials. Pre- and post-induction surveys for pediatric doctors showed an increase in confidence among responders in performing practical procedures, prescribing intravenous fluids, and medication prescribing [16]. This study, in particular, highlights the importance of not relying on one method of induction, and considering a healthy mix of practical and non-practical induction elements.

We conclude from the literature review that an ED handbook is likely to help increase the knowledge and confidence of doctors starting their emergency department rotation. For maximum benefit, it should include relevant medical protocols, guidelines, referral pathways, bleeps, and contact details. It should also be accessible online and offline, be accessible on all smartphones and PCs, and be present as a physical hardcopy in the ED doctor’s office for quick reference. The size of the file should also be small enough to be shared with juniors via email and be accessible/downloadable from the hospital intranet.

## Materials and methods

Data collection

This change was observed by undertaking surveys before and after the introduction of the ED Handbook. A Plan-Do-Study-Act (PDSA) cycle was initiated. Four surveys were done across the course of the quality improvement project (QIP) from August 2022 to March 2023.

Survey 1 was the baseline pre-intervention survey done in the August 2022 Junior Doctor Induction before the ED Handbook was created. It collected responses from new junior doctors in the department about their satisfaction with the induction and their level of confidence to start their new jobs. Survey 2 was done in November 2022 on the same cohort of juniors to assess the demand for an ED handbook and what content they would like to include in the handbook. Survey 3 was done in the February 2023 Junior Doctor Induction for a subsequent cohort of six new junior doctors. In this induction, the digital ED Handbook was introduced to them and a tutorial session on how to use it was delivered. The survey collected information about their satisfaction with the induction and their level of confidence to start their new jobs. Survey 4 was done in March 2023, one month after the handbook was introduced to the same cohort of doctors that joined in February 2023. It assessed how useful the handbook was in increasing their knowledge and confidence after one month of use, their satisfaction with the induction, and whether they recommended the handbook for future cohorts.

The surveys were created on Google Forms and sent to junior doctors working in ED at the time of the survey by email. This allowed for the data to be easily collected and meant it was easier for respondents to respond at a time that was convenient to them. The data produced from the responses were then put into a Microsoft Excel (Redmond, WA: Microsoft Corp.) file to create the pie charts in the results section.

Intervention strategy

Following the results of survey 2 which showed that there was a demand among juniors for an ED handbook, the Quality Improvement Department at Queen Elizabeth Hospital, King’s Lynn (QEHKL) gave the project full approval to proceed in January 2023. Material for the ED handbook was collected, organized, edited, and referenced by the project members which included senior doctors and colleagues from the IT department. It was introduced in the February 2023 ED Junior Doctor Induction for the new cohort joining in February 2023. This cohort received a copy of the electronic ED Handbook by email one week before February 2023 induction so that they could familiarize themselves with the contents of the handbook.

An interactive tutorial session explaining how to use the handbook was carried out during the induction using quizzes to help them get used to navigating the handbook day to day. The handbook was made easily accessible from any smartphone and the hospital intranet with help from the IT department at QEHKL. A physical version of the handbook was also made available and put in the ED main doctor’s office for those who preferred a physical copy.

Target population

The target population comprised junior doctors working in the ED at QEHKL. Our target audience is doctors at the tier 1 and tier 2 levels. The responders included trainees and non-trainees. Tier 1 doctors comprised of the Foundation Year 2 (FY2), Senior House Officers (SHO), GP-Speciality Trainees (GPST), and Acute Care Common Stem (ACCS) trainees. Tier 2 is comprised of EM Speciality Trainee Year 3 (ST3) doctors.

The number of responders varied between each survey with there being nine in Survey 1, 11 in Survey 2, six in Survey 3, and nine in Survey 4. This variability was due to some non-responders and some doctors joining in at later times. We aimed to have a 100% response rate. All the surveys were anonymous. Across the surveys, most responders were tier 1 doctors. Responders in Surveys 1 and 2 were from the same cohort and were the same individuals. Responders for Surveys 3 and 4 were from a subsequent cohort and they were the same individuals.

Ethical considerations

After Survey 2, we presented the results of our findings alongside the proposed handbook to the ED clinical governance team which consists of the ED clinical lead, ED governance lead, other ED consultants, and senior nurses who approved the handbook. There were no conflicts of interest. The contents of the handbook were reviewed carefully for accuracy and guideline expiry dates. Any external guidelines were clearly referenced.

## Results

Primary objectives

**Figure 1:**
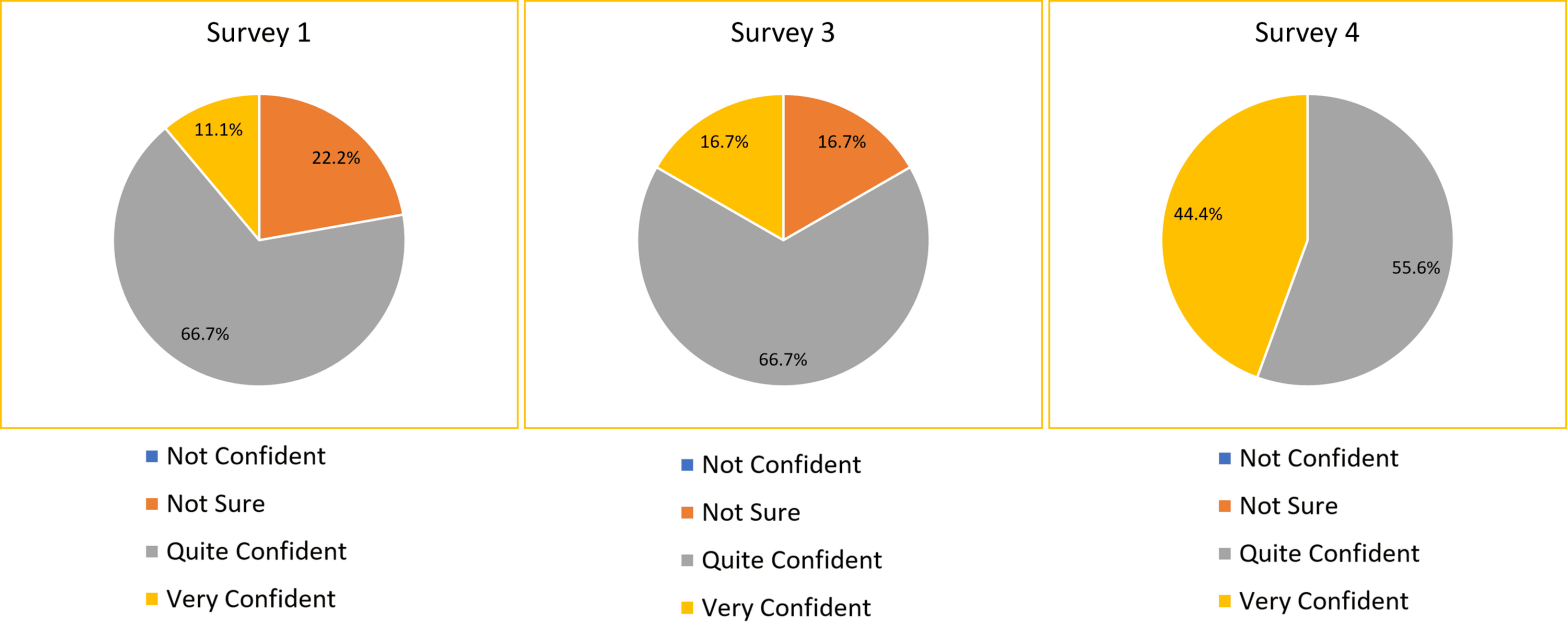
The pie charts show ED junior doctors perceived confidence before the handbook’s introduction (Survey 1), after the handbook’s introduction on day one (Survey 3), and after one month of handbook use (Survey 4). This relates to the primary objective. Candidates were asked, “How confident are you to proceed into your new role after the induction?”

In Survey 1 prior to the handbook being introduced, 77.8% of the respondents responded that they felt either "quite confident" or "very confident" to start their ED rotation. When the handbook was introduced, there was an increase in this figure to 83.4% in Survey 3. After one month of handbook use, this increased further to 100% in Survey 4 (Figure 1). In Survey 1 before introducing the handbook only 11.1% of responders said they felt “very confident” to proceed with their new roles. This figure rose to 44.4% by Survey 4 (Figure 1).

**Figure 2:**
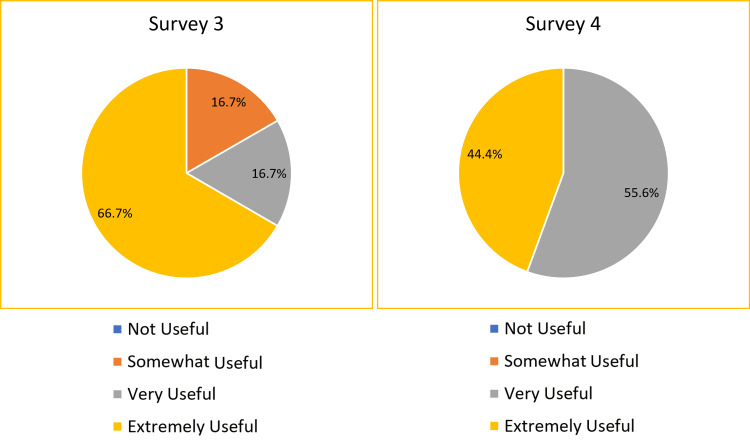
The pie charts show how useful the ED Handbook was in increasing the confidence of new junior doctors in ED on day one of the initial induction (Survey 3) vs after one month of handbook use (Survey 4). This relates to the primary objective. Candidates were asked, “How useful do you think the ED Handbook has been to increase your confidence as a new doctor in ED?”

In Survey 3, on day 1 of the handbook’s introduction, 77.8% of the responders found the ED Handbook to be either “very useful” or “extremely useful” in increasing their confidence (Figure 2). In Survey 4, after one month of handbook use, 100% of the responders found the ED Handbook to be either “very useful” or “extremely useful” in increasing their confidence (Figure 2).

**Figure 3:**
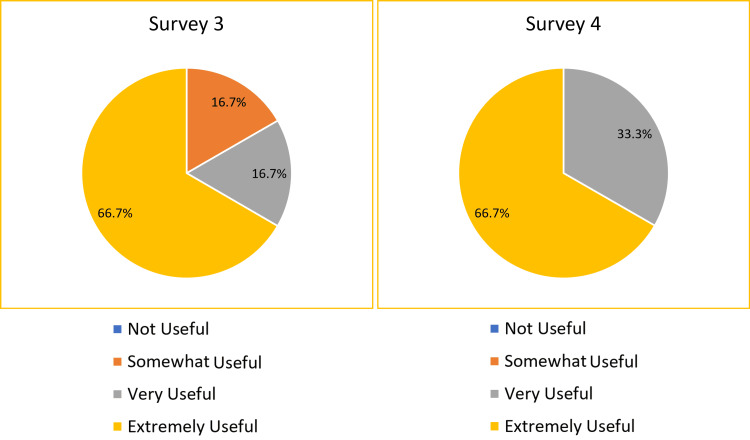
The pie charts show how useful the ED Handbook was in increasing the perceived knowledge of new junior doctors in ED on day one of the induction (Survey 3) vs after one month of handbook use (Survey 4). This relates to the primary objective. Candidates were asked, “How useful do you think the ED Handbook has been to increase your knowledge as a new doctor in ED?”

In Survey 3, on day 1 of the handbook’s introduction, 77.8% of the responders found the ED Handbook to be either “very useful” or “extremely useful” in increasing their knowledge (Figure 3). In Survey 4, after one month of handbook use, 100% of the responders found the ED Handbook to be either “very useful” or “extremely useful” in increasing their knowledge (Figure 3). Of note, 66.6% of them responded that they used the handbook either “daily” or “several times per week” in the first month in Survey 4.

Secondary objectives

**Figure 4:**
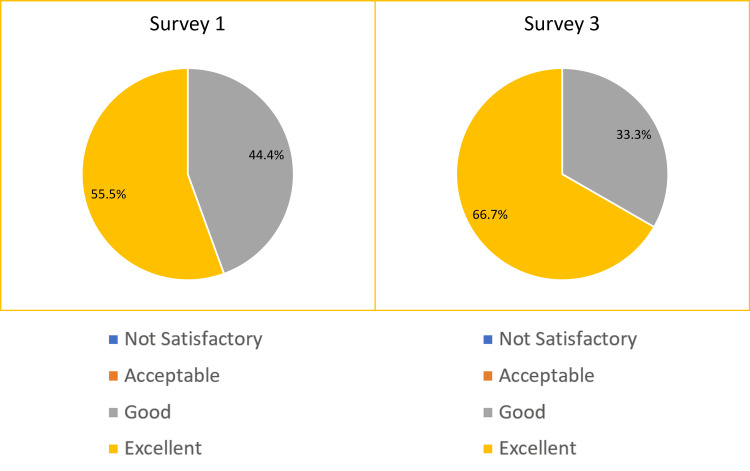
The pie charts show ED junior doctors’ satisfaction rating of the induction program before (Survey 1) vs after the introduction of the ED Handbook (Survey 3). This relates to the secondary objective. Candidates were asked, “How satisfied were you with the content of the ED Junior Doctor Induction Programme?”

In Survey 1, before the introduction of the ED Handbook, 55.6% of responders said that the content of the ED induction was “excellent.” This figure increased to 66.7% in Survey 3 after introducing the ED Handbook (Figure 4). Both surveys had 100% ratings of either “very good” or “excellent.” Additionally, 100% supported the inclusion of the ED Handbook for the next induction.

## Discussion

The induction/orientation before the ED Handbook consisted of small tutorials that covered handovers, bleeps, electronic systems, referral pathways, brief lectures on common presentations, and intermediate life support simulations. The induction program would take two to three days. While there were some brief handouts, most of the information was delivered verbally. The amount of information could be overwhelming and there was not a document that summarized all of the information in one place.

The project was able to find that the introduction of an ED handbook could significantly increase the confidence and knowledge of junior doctors in their first ED rotation at Queen Elizabeth Hospital, King's Lynn. It may also increase the satisfaction ratings of the content of the inductions. It also garnered 100% support for inclusion in the inductions of future cohorts of doctors joining the department.

The results also suggest that the juniors engaged with the material well. A total of 66.6% used the handbook either “daily” or “several times per week.” This reflects the relevance of the content to their daily practice, the ease of navigation, user-friendliness, and compatibility with all smartphones. Furthermore, the juniors were asked beforehand about the content they would expect in the handbook, and their responses directed us to decide what topics to include and exclude.

The handbook was also designed to be a glanceable “touch and go” resource that is painless to read and quick to go through. This was achieved by emphasizing the use of diagrams as opposed to lengthy text. The contents page was linked to specific pages in the digital booklet, so that pressing on a topic would take you directly to the intended page. This helped the user avoid spending time scrolling through unintended topics to get to the information they require, thus making the handbook easy to navigate. The use of electronic surveys provided a whole host of advantages. These included being able to easily collate the responses and the data itself being secure. If there were any errors in the questions, these could be easily amended before distribution.

One of the strengths of the study is the high respondent rate, which did not drop below 50%. The research objectives were set as Specific, Measurable, Achievable, Relevant, and Time-bound (SMART), allowing us to assess the success of the QIP. Additionally, the study design facilitated a direct comparison between pre-intervention and post-intervention surveys.

One of the limitations of the project lies in the small study populations. Overall, this is a limitation of the size of the hospital and the number of juniors rotating into ED at any given term. This could mean that the positive results we garnered from the handbook might not be replicable in larger hospitals. Another limitation relies on the QIP only being carried out in one hospital. A multi-center approach would enable us to see if the positive results at QEHKL were replicable elsewhere. The number of responders was not the same across all surveys due to some doctors being out of sync in their rotations with others. This predisposes the data to some non-response bias.

One of our considerations was to introduce the ED Handbook in the form of a smartphone app. Clare et al. explored introducing an app called “Induction” for referrals and bleep numbers [12]. There were positive impacts of introducing the app such as greater ease in requesting investigations and finding guidelines. There are positive impacts in terms of its portability, ease of use, security, and being freely downloadable. However, as noted by the authors, there were challenges noted in promoting awareness of the app’s availability. There are also time-consuming compatibility issues that arise when trying to install the app on older smartphones. This could have a negative impact on its accessibility. We do not currently have the funding or experience to make it into a smartphone app.

## Conclusions

We are confident that the introduction of the ED Handbook has created a tool that better supports and improves junior doctors' induction into ED through our surveys. The handbook was received with much positivity and appreciation by the consultants in the department, the medical director, and the quality improvement department.

We are actively seeking feedback for the current version. We will be working with the IT department again to put the ED Handbook on the desktop of the computers in our ED for easy access. Other departments within the hospital have expressed interest in emulating the handbook for their specialties to potentially achieve the same benefits. We have been given approval by the clinical governance team in March 2023 to use the handbook for future ED junior doctor inductions. A second edition was also released.
